# Characteristics of Newly Developed Extruded Products Supplemented with Plants in a Form of Microwave-Expanded Snacks

**DOI:** 10.3390/ma14112791

**Published:** 2021-05-24

**Authors:** Katarzyna Lisiecka, Agnieszka Wójtowicz, Marek Gancarz

**Affiliations:** 1 Department of Thermal Technology and Food Process Engineering, University of Life Sciences in Lublin, Głęboka 31, 20-612 Lublin, Poland; katarzyna.zelizko@gmail.com; 2 Institute of Agrophysics Polish Academy of Sciences, Doświadczalna 4, 20-290 Lublin, Poland; m.gancarz@ipan.lublin.pl

**Keywords:** plant material, fresh carrot, extrusion-cooking, microwave expansion, snacks, physical properties, color, texture

## Abstract

The following research focuses on the possibility of applying fresh plant material as a carrot pulp to supplement newly developed extruded products in the form of pellets and microwave-expanded snacks. Fresh carrot pulp, as a valuable vegetable ingredient, was used in the amount of 2.5 g/100 g to 30.0 g/100 g in a potato-based recipe. The snack pellets were processed via extrusion-cooking, using a single-screw extruder with a plasticizing unit L/D = 18, and the use of variable screw speeds. The produced pellets underwent microwave expansion to limit the fat content, so as to produce ready-to-eat (RTE) snacks. The pellets and snacks were tested for nutritional value, as well as for selected quality features: physical properties, structure, pasting characteristics, and texture profile, and PCA analysis and a correlation matrix were performed on the obtained results. Microwave expansion of pellets increased the total phenolic content, the antioxidant activity, water absorption index and lightness of snacks, but decreased the bulk density and setback values of the expanded products when compared to pellets. Generally, we found that it was possible to use up to 30.0 g/100 g of fresh carrot pulp with a positive effect on nutritional value, and without negative effects on the physical properties of extruded products. Both the extrusion-cooking and microwave expansion can minimize the negative impact on plant materials, due to the short processing time.

## 1. Introduction

Plant materials are recognized as the most important sources of the nutritional components that are necessary in the daily human diet [1]. Among the positive characteristics of plants, their antioxidant properties are the most underestimated [2]. Indeed, the extracted and concentrated biologically active compounds from plants can have an impact on the prevention or help of many pathological conditions [3].

Carrots are a valuable source of carotenoids and fiber [1]. A diet rich in dietary fiber prevents the occurrence of diabetes, esophageal hernia, obesity, etc. [4]. This vegetable is also an extremely important source of vitamin A, due to the presence of *β*-carotene [5]. Additionally, carrots are rich in vitamins C, B_1_, B_2_, B_6_, and B_9_ as well as the mineral components, calcium, magnesium and phosphate. The carotenoids of carrots, in possessing antioxidant properties, contribute to cancer and cardiovascular disease prevention [6,7]. Moreover, the consumption of carrots is recognized for decreasing cholesterol levels, and positively influencing the whole-body condition [1,8]. Vitamin C prevents infection and supports the absorption of non-heme iron. B_1_ has a positive influence on the nervous system, while B_2_ is necessary for cellular respiration and the formation of red blood cells. Vitamins B_6_ and B_9_ protect against disease and heart attacks [8]. The overall vitamins and minerals provided by the consumption of carrots also reduce the risk of chronic diseases, cancer and stroke [9]. Therefore, fresh carrot pulp could be a potential nutritionally valuable additive for enhancing the nutritive value of snack foods. This is especially relevant today, when consumers are looking for healthier alternatives with pro-health properties to traditional snack foods [10].

Extrusion-cooking is an economic HTST (high temperature–short time) processing technique that is used in the manufacture of a wide range of cereal- and pulse-based processed puffed foods [11]. As an alternative to ready-to-eat (RTE) extrudates, third-generation half product snack pellets are usually generated through so-called cold extrusion. Such pellets require an extra manufacturing step after pressure-thermal treatment, that is, they must undergo an expansion process. Traditionally, pellets are expanded by frying in deep oil, but other expansion methods have become more popular, i.e., hot air toasting or microwave heating to limit the fat content in RTE snacks [12].

The properties of extruded snack pellets, with the addition of fresh carrot pulp, expanded by microwaving has not yet been subject to study. Plant material such as fresh carrot pulp seems to be a valuable source of biologically active components, as well as a natural colorant. Moreover, as was assumed by Lisiecka and Wójtowicz [13], the application of fresh vegetable pulp is an efficient method to limit water and energy requirements during processing. Therefore, the present study is aimed at investigating the influence of the addition of varied fresh carrot content and variable screw speeds during processing on selected features of newly developed pellets and microwave-expanded snacks.

## 2. Materials and Methods

The basic raw materials were potato flakes (PF), potato grits (PG) and potato starch (PS,) provided by Pol-Foods Sp. z o. o. (Prostki, Poland). Ordinary carrot (*Daucus carota*) was bought at the local market in Lublin, Poland. The carrots were carefully washed with tap water, dried using paper towels, and ground by employing an LMN-100 laboratory knife mill TestChem (Radlin, Poland) for granulation below 1 mm, to achieve fresh carrot pulp (FCP). The moisture content of all raw materials was checked according to Lisiecka and Wójtowicz [13].

### 2.1. Pellet and Snack Preparation

The composition of the control sample was 25 g/100 g potato flakes, 25 g/100 g potato grits, and 50 g/100 g potato starch, according to the recipe. Other blends were supplemented with fresh carrot pulp (FCP) at a range varying from 2.5 g/100 g to 30.0 g/100 g. The vegetable additive replaced the normal amount of potato starch in the prepared blends. All components were ground together, utilizing a laboratory knife mill with 0.4 mm sieve openings, to achieve blends with a particle size below 400 µm before processing. The final moisture content of each blend (33%) was obtained by spraying the blended components with the appropriate amount of tap water and then mixing for 15 min. The amount of water was calculated based on the water content in all blend components, as described by Lisiecka and Wójtowicz [13], and thus, the application of fresh plant material significantly limited the water needed for processing.

**Figure 1:**
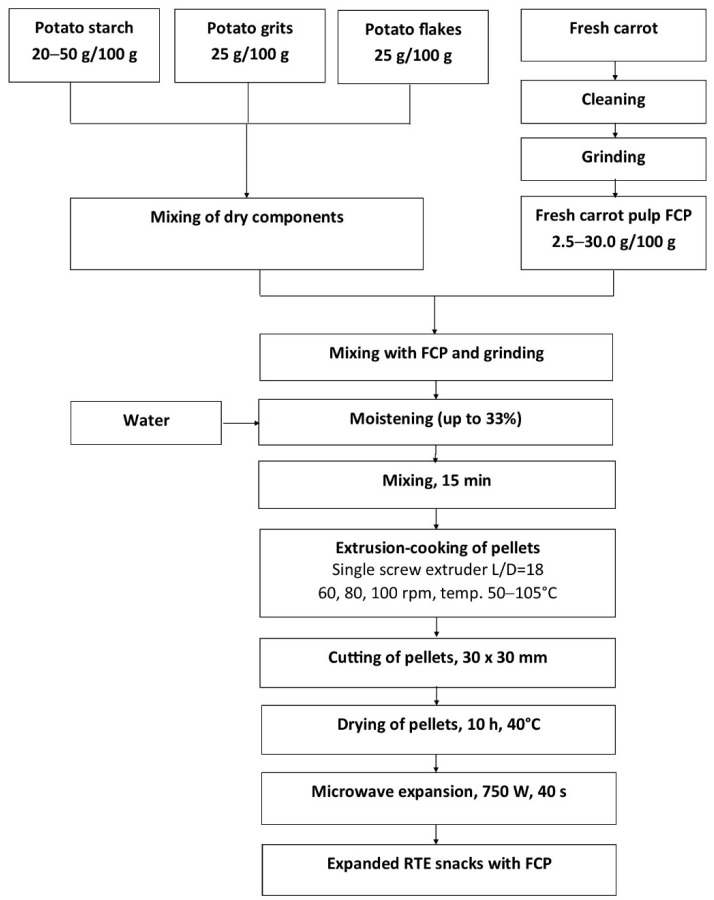
Diagram of the production process of pellets and snacks.

The blends were extruded by using a single-screw extruder type TS-45 (ZMCh Metalchem, Gliwice, Poland) with a plasticizing unit profile of L/D = 18, which incorporated an intensive glycol cooling section (Figure 1). The temperature range in the individual extruder sections was as follows: dosing section, 80–85 °C; plasticizing section, 90–105 °C; cooling section, 50–65 °C; forming die, 60–75 °C. Screw speeds ranging from 60 rpm to 100 rpm were applied to obtain snack pellets. The pellets were shaped as a dough strip using a 30 × 0.4 mm flat forming die, and then cut to approx. 30 × 30 mm squares. The extruded snack pellets were dried in a laboratory shelf dryer at 40 °C by 10 h until the moisture content reached 11%. The dried pellets were subsequently placed in sealed foil bags and stored until the final processing. The pellets were expanded to ready-to-eat (RTE) snacks by microwave treatment (AVM-914/WH Philips/Whirlpool, Sweden). The power of the microwave was 750 W, and the optimal time of treatment (40 s) was selected experimentally.

### 2.2. Chemical Properties

#### 2.2.1. Solvent Extraction Procedure

Ground pellets or expanded snacks (0.5 g), with a granulation below 300 µm, were extracted in double by using 5 mL of 50% methanol (Avantor Performance Materials, Gliwice, Poland). The resulting extracts were shaken with a Multi RS-60 rotator (Biosan, Riga, Latvia) operating at 4200× *g* by 30 min. The homogenate was then centrifuged using a T24D-type centrifuge (Medizintechnik, Leipzig, Germany) at 4200× *g* for 10 min at 4 °C. The collected supernatants of methanol extracts were used for further analysis [14].

#### 2.2.2. Total Phenolic Content (TPC)

Analysis of the total phenolic content in samples was prepared according to Singleton et al. [15], modified by Lisiecka et al. [16]. Here, 0.05 mL of 50% methanol, 0.1 mL of H_2_O and 0.4 mL of Folin–Ciocalteu reagent (at a ratio of 1:5 with distilled water) was mixed with the 0.05 mL of extract. After 3 min, 2 mL of 10% sodium carbonate was added to the mixture and shaken for 1 min. Of note, 0.05 mL of the tested extract was replaced by 50% methanol in a reference sample. The absorbance was measured at λ = 725 nm, using a spectrophotometer (Model 9423, Alt, GA, USA), and the results were expressed as gallic acid equivalents (GAE) in mg/g of dry weight (d.w.).

#### 2.2.3. Scavenging Activity on 1,1-Diphenyl-2-picrylhydrazyl (DPPH) Radical

In brief, in order to obtain a working solution, DPPH (1,1-diphenyl-2-picrylhydrazyl) radical was diluted in methanol to reach UV–VIS absorbance 0.7 at 515 nm. Subsequently, 2.5 mL of DPPH radical, with a concentration of 0.2 mM, and 0.1 mL of the sample were mixed and kept for 30 min in a dark place, before measurement via spectrophotometer (Model 9423, Alt, GA, USA). Scavenging activity was calculated by using the formula according to Oniszczuk et al. [17], based on the method of Blois [18].

### 2.3. Physical Properties

#### 2.3.1. Bulk Density, Volumetric Expansion Index and Structure

The bulk density (BBD) was specified as the mass of the sample, divided by the occupied volume of the cylinder (Lisiecka & Wójtowicz, 2019) [19]. The volumetric expansion index (VEI) was calculated as the ratio of the density of pellets and snacks after expansion. The pellet and microwave-expanded snack structures were observed with an optical microscope (MBL3000T, mikroLAB, Poland), with magnification ×10 for the surface and ×100 for the cross-section.

#### 2.3.2. Water Absorption Index (WAI) and Water Solubility Index (WSI)

0.7 g of the sample was mixed with 7 mL of distilled water for 10 min and then centrifuged using a T24D-type centrifuge (Medizinetechnik, Leipzig, Germany) with a speed of 12,577× *g* for 10 min. The gel was then separated from the filtrate. The water absorption index (WAI) was calculated as the ratio of gel weight to the weight of the dry sample. The filtrate was dried at 110 °C until the water had completely evaporated. Water solubility index (WSI) was determined as the ratio of solids by weight in the supernatant after drying to the dry weight of the sample. WAI and WSI were expressed in g/g and %, respectively [20].

#### 2.3.3. Color Measurements

The color profile was evaluated using an NR20XE Large Aperture Precision Colorimeter (Shiyan, Shenzhen, China). The color coordinates in the CIE-Lab scale indicated L*, a* and b* described the brightness from 0 to 100, the balance between redness (+) and greenness (−), and the balance between blueness (−) and yellowness (+), respectively. The *ΔE* was calculated as the total color change index [10].

### 2.4. Pasting Properties

The pasting properties of pellets and snacks were tested as described by Mitrus et al. [21], using the Brabender Micro Visco-Amylo-Graph (Brabender GmbH & Co. KG, Duisburg, Germany). Accordingly, 10 g of the ground sample was dispersed in 100 mL of distilled water. The experiment was conducted at a speed of 250 rpm and a sensitivity of 235 cmg. During all measurements, the following test profile was used: heating (30–93 °C) with a temperature gradient of 7.5 °C/min, holding (93 °C for 5 min), cooling (93–50 °C) with a temperature gradient of 7.5 °C/min, holding at 50 °C for 1 min. Selected pellet or expanded snack characteristics that were assessed included: peak viscosity (PV); hot paste viscosity (HPV); cold paste viscosity (CPV); breakdown (BD) as the difference between PV and HPV; and setback (SB) as the difference between CPV and HPV, using the Brabender Viscograph software (version 4.1.1) application.

### 2.5. Texture Measurements

Textural characteristics were examined using a Zwick/Roell BDO-FB0.5TH (Zwick GmbH & Co. KG, Ulm, Germany) testing machine equipped with a 0.5 kN working head. The cutting force (CF) of the pellets was evaluated using a Warner–Bratzler shear device with a cutting test speed of 500 mm/min. Texture measurements of the microwave-expanded snacks were determined a maximum of 10 min after expansion using the same apparatus, with a compression test undertaken via the use of a Kramer shear cell with a working head test speed of 100 mm/min, until the snack was completely broken down. Hardness (H) was determined as the highest peak generated by the software during compression. Crispness (CR) was evaluated as the difference of force between the first significant peak force and the first minimum force following the first peak. Fracturability (F) was the first significant force peak during the compression cycle [22].

### 2.6. Statistical Analysis

Statistical analysis of the results was completed using Statistica 13.3 software (StatSoft, Tulsa, OK, USA). Response surface methodology was used to show the effect of multiple variables using a second-order polynomial model [5]. Homogeneous groups were determined by ANOVA using the Tukey post-hoc test at α = 0.05. The correlation matrix was formed of dependent features and by grouping variables using Pearson’s coefficients. The similarity of pellets and snacks in terms of their analyzed features was based on principal component analysis (PCA). The PCA data matrix for the statistical analyses of the pellet results had 15 columns and 21 rows. However, the PCA data matrix for the statistical analyses of the results obtained for the snacks had 17 columns and 21 rows. The input matrix was scaled automatically. The correct number of main components obtained in the analysis was determined based on Cattell’s criterion.

## 3. Results and Discussion

### 3.1. Total Phenolic Content (TPC) and Antioxidant Activity of Snacks

The release of phenolic acids can be related either to the selective heating of a number of individual phenolic acid compounds in the microwave field, or to the physical forces acting between the phenolic acids and the plant matrix [23]. Table 1 shows the total content of phenolic compounds (TPC), as well as the antioxidant activity (AA) of pellets and of the microwave-expanded snacks supplemented with plant material.

We saw that the microwave treatment increased the total content of polyphenols (at least by 33%) and the ability to scavenge free radicals (increased by at least 47%) in snacks compared to pellets. However, an insignificant effect of increased screw speed applied during the processing of samples was observed with the same amount of fresh carrot pulp, both for pellets and expanded snacks. Additionally, there was a positive correlation between the content of the additive and of the TPC and the AA in pellets (r = 0.97 and 0.91) and snacks (r = 0.91 and 0.94), respectively. A strong positive correlation was also noted between the TPC of pellets and the TPC of snacks (r = 0.91), and between the TPC of snacks and the ability to scavenge free radicals in snacks (r = 0.94). Furthermore, it was noticed that the addition of fresh carrot pulp in the amount of 10.0 g/100 g and 20.0 g/100 g of pellets, as well as of 30.0 g/100 g and 7.5 g/100 g in the case of microwave-expanded snacks, significantly influenced the TPC and DPPH values, respectively, in comparison with the control sample. It is likely that microwave energy was efficiently delivered to materials by interacting with the electromagnetic field, providing rapid energy transfer. This effect can cause structural and morphological changes to plant tissues, making them more accessible to solvent extraction [24,25].

Reports exist in the literature about microwave-expanded corn pellets with orange and powdered milk additions, in which an increase in total polyphenols after microwave treatment at 1450 W has been described [26]. Hayat et al. [23] also observed an increase in the content of polyphenols, and an enhancement of the antioxidant properties of extracts made from the citrus mandarin peel, after microwave treatment. Both groups of researchers noticed that the microwave power and the treatment time affected the phytochemical properties. Here, increased power and elongated time were found to be responsible for the degradation of some phenolic compounds. In contrast, the increase in antioxidant activity that corresponded with the increase in exposure time and microwave power can be attributed to the increase in free phenolic compound fractions—which demonstrate a greater antioxidant effect than do the bounded forms.

### 3.2. Physical Properties

**Figure 2:**
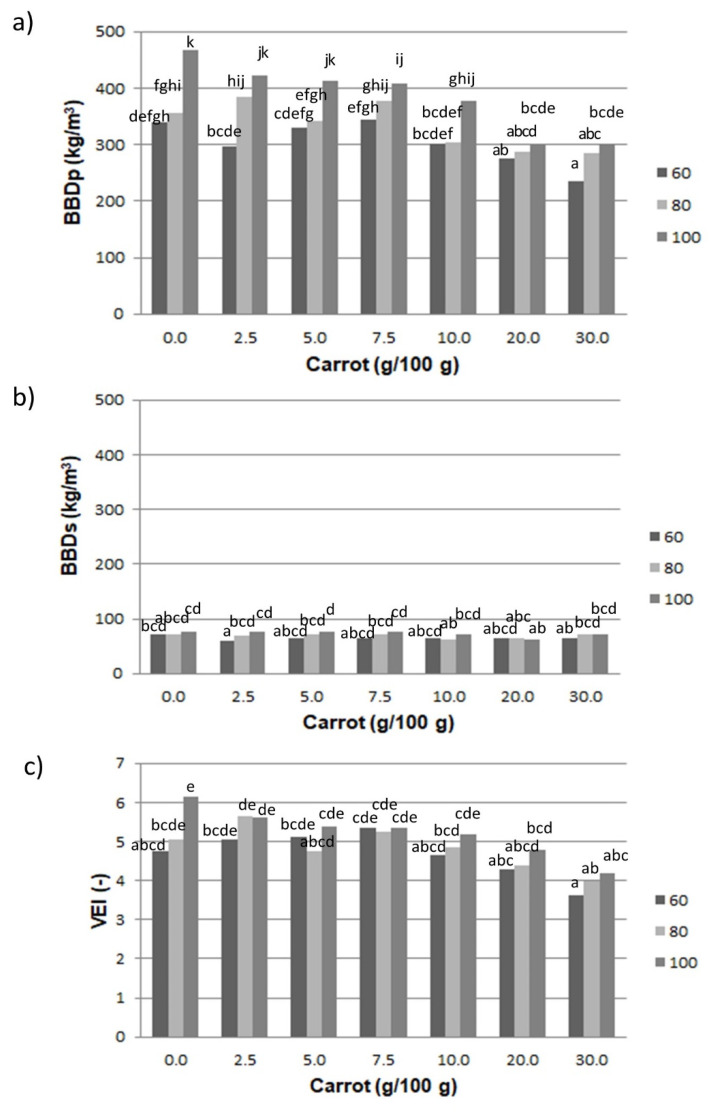
Results of the physical properties of extrudates supplemented with fresh carrot pulp, processed at variable screw speeds: (**a**) the bulk density of pellets, (**b**) the bulk density of snacks, (**c**) volumetric expansion index of snacks. ^a–k^—means indicated with similar letters in each figure do not differ significantly at α = 0.05.

The bulk density (BBDp) of the tested pellets and expanded snacks (BBDs), and the volumetric expansion index (VEI), are shown in Figure 2. In the case of pellets, an increase in BBD was noticed with the increase in screw speed at each level of the fresh carrot pulp addition. The range of the pellets’ BBDp was from 236.04 kg/m^3^ (pellets with 30.0 g/100 g of additive content at minimum rpm) to 466.48 kg/m^3^ (control sample at maximum rpm). Snacks expanded by the microwaving of these pellets were characterized by the smallest and the highest values of the expansion index, respectively (Figure 2c), but the differences were small, and insignificant differences were found depending on both the vegetable content and the screw speed applied during extrusion. It suggests a similar expansion of vegetable supplemented snacks via microwaves, which was also confirmed by the results obtained for the texture profile of snacks. 

As expected, there was a strong positive correlation between the pellets’ density and the VEI (r = 0.90). A negative correlation, however, was observed between the amount of additive and the BBDp of pellets (r = −0.71) and the VEI (r = −0.83). On the other hand, a positive correlation was noted between the screw speed and the density of pellets and snacks (r = 0.58 and 0.65, respectively). The BBDp of pellets was also positively correlated with the density of the snacks (r = 0.74). Moreover, there were some significant differences found between the pellets’ bulk density values between 60 rpm and 100 rpm, except at the 20.0 g/100 g level.

Microwave-expanded snacks with the addition of fresh carrot pulp had BBD values ranging from 58.79 kg/m^3^ (snacks with 2.5 g/100 g of carrot pulp at maximum rpm) to 76.93 kg/m^3^ (snacks with 5 g/100 g vegetable content at maximum screw speed). The bulk density of soybean-based RTE extrudates also increased with an increasing rotational screw speed during extrusion [27], because of the high temperature-induced starch gelatinization, which makes it possible to obtain snacks with a lower density despite the use of increasing rpm, as shown by the production of brown rice extrudates. The low density is influenced by the breakdown of the protein and starch matrix under high shear forces [28]. Since the production of third-generation expanded snacks from starchy pellets involves the use of lower temperatures to avoid the boiling of the water present in the raw materials, and thus the direct expansion of the pellet strip, this outcome is most likely connected with the higher density and viscosity of the dough at increased applied rpm during processing, as is indicated by significant differences in the pellets’ bulk density when processed at the lowest and the highest screw speeds (Figure 2a).

**Figure 3:**
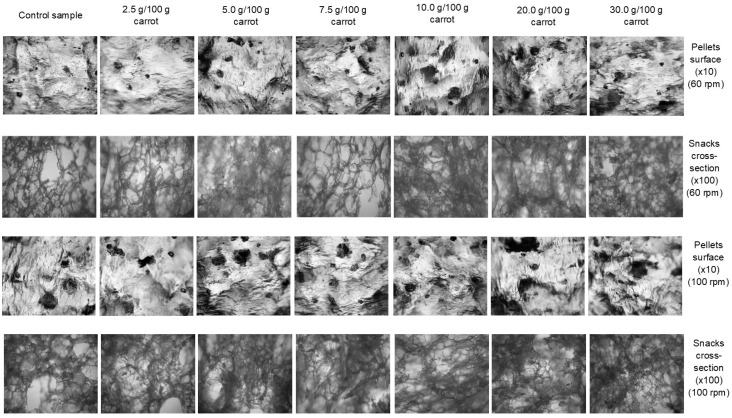
Microscopic structure of pellet and microwave-expanded snack surfaces (×10 magnification), and cross-section (×100 magnification). Herein, the structure depends on the content of the fresh carrot pulp addition (in columns) and screw speed during processing (in rows).

Figure 3 shows the structure of fresh carrot pulp-supplemented pellet surfaces (×10 magnification), and cross-sections of the microwave-expanded snacks (×100 magnification), respectively. Microscopic observations confirmed the compact and glassy surface of pellets, indicating a good gelatinization of the extruded samples. Such an effect became more evident as the carrot pulp addition increased, and with this increase, vegetable particles could be seen on the surfaces and in inside the structures of the extruded pellets. Pellets processed at a higher screw rpm were less smooth on the surface, especially when high amounts of vegetables were added, as shown in Figure 3. On observing the cross-section of microwave-expanded snacks, we found a porous inside structure with empty cells that increased in quantity, yet had dimensions that decreased in size with the increased level of fresh carrot pulp in the recipe. Nevertheless, all the tested snack pellets supplemented with fresh carrot pulp successfully underwent microwave expansion. This increased level of vegetable additive made the inside structure denser and limited cell size, as was confirmed by the VEI results.

The WAI measures the amount of water absorbed by the starch and can be used as an indicator of the degree of starch gelatinization. The WSI value describes the degradation of molecular components after technological treatment, which is measured by the amount of soluble polysaccharides released from the product, and indicates the degree of starch conversion during the extrusion-cooking process [28]. Table 2 presents the values of the WAI and WSI for the tested pellets and microwave-expanded snacks. The maximum value of WAI for pellets and snacks was 4.90 g/g and 5.61 g/g, respectively, for pellets with 5.0 g/100 g of fresh carrot pulp content extruded at 80 rpm and for expanded snacks with 20.0 g/100 g of additive, processed at maximum screw speed. A minimum WAI value was observed for pellets with 7.5 g/100 g of vegetable content, and snacks with 30.0 g/100 g of the additive, processed at the lowest rpm. However, a significant difference in the WAI value was only noticed in the extreme values of the pellets, compared to their controls, produced at identical rpm. The WSI values ranged from 1.42% to 5.69% for pellets produced at 60 rpm, with the addition of 5.0 g/100 g and 30.0 g/100 g of fresh carrot pulp, respectively. The microwave treatment increased the WSI of the snacks, compared to the pellets. The WSI of the supplemented snacks ranged from 2.34% (5.0 g/100 g of carrot processed at 80 rpm), to 10.48% (20.0 g/100 g snacks extruded at 100 rpm). At the same time, it was noticed that the maximum WSI values of the sample of pellets and snacks differed significantly from the other samples containing less than 7.5 g/100 g and 20.0 g/100 g of additive, respectively, which was not dependent on the rotational speed of the extruder screw.

According to the literature, the values of the WAI and WSI depend on two main variables. The first variable includes factors directly related to the properties of raw materials, their composition, the pre-treatment, and the method of their grinding. The second includes temperature, humidity, screw speed, configuration and compression ratio of the screw, and the dimension and configuration of the forming die [29]. In our study, a positive correlation was found between the amount of the additive and the WSI value of pellets and expanded snacks (r = 0.53 and 0.67, respectively). The WSI index of pellets and snacks was also negatively correlated with the BBD results (r = −0.59 and −0.60, respectively), as well as with the VEI (r = −0.60 for both products). In the case of WSI, there is a positive correlation between the WAI of pellets and snacks. Silva et al. [30] observed that low moisture content of extruded blends and high sucrose content lead to an increase in the WSI. The low molecular weight and the addition of sugars tended to increase the content of soluble solids. The unambiguous effects of both vegetable content in the recipe and screw speeds applied on WAI and WSI were observed, as confirmed by the indication of homogenous groups for these results.

Table 3 shows the values of the L*, a*, and b* coordinates, as well as the *ΔE* index of pellets and microwave-expanded snacks that have been supplemented with fresh carrot pulp. The microwave treatment increased the lightness of the expanded snacks in relation to the pellets, independently of the content of the additive and the screw speed applied during processing. This change in color was attributed to the loss of the compact structure and, hence, a change in light passing through the pellets after microwaving. Both the pellets and expanded snacks showed a greater redness and yellowness with the increase in the percentage of fresh carrot addition. The *ΔE* index revealed, however, that there were significant changes only in the color of the RTE snacks. We believe that the color of both pellets and snacks was affected by the carotenoids contained within the fresh carrot, used as an additive. Carotenoids contribute to the formation of red, orange and yellow colors [31]. However, in the literature, color changes are also attributed to browning reactions, e.g., caramelization, Maillard reactions, or pigment degradation, which sometimes accompany high-temperature treatment [32]. Moreover, the addition of fresh carrot pulp, which is rich in simple sugars, may also affect the intensity of browning during microwave heating if more of the vegetable was used.

The L* coordinate of the pellets showed a strong negative correlation with the amount of additive (r = −0.83), as well as with a* (r = −0.87) and b* (r = −0.80) pellet coordinates. It also showed a positive correlation with the VEI (r = 0.89). Moreover, the a* coordinate of the pellets showed a strong positive correlation with the amount of additive (r = 0.97), and the pellet and expanded snack TPC (r = 0.97 and 0.89, respectively), as well as the AA of the pellets (r = 0.84) and snacks (r = 0.91), the b* coordinates of the pellets (r = 0.85), and a* and b* values of the expanded snacks (r = 0.90 and 0.91, respectively). In contrast, a negative coefficient was found between a* and VEI (r = −0.83). In addition, the b* coordinate of pellets was positively correlated with its counterpart, found in the expanded snacks (r = 0.80). The paleness of the microwave-expanded snacks was negatively related only to the redness of the snacks (r = −0.81). The a* and b* coordinates of supplemented snacks were, respectively, positively related to the amount of additive (r = 0.92 and 0.91), the TPC of pellets (r = 0.92 and 0.90), the AA of snacks (r = 0.92 and 0.87), and were strongly related to each other (r = 0.97). Generally, it was noticed that the minimum L* values of the pellets with 30.0 g/100 g of carrot extruded at 80 rpm differed significantly from the other samples. In the case of the pellets, it was noticed that the addition of more than 5.0 g/100 g of fresh carrot pulp significantly affected the value of the a* coordinate, while in the case of snacks, this relationship was already visible above 2.5 g/100 g. On the other hand, in the case of b*, it was noted that the addition of more than 10.0 g/100 g and 20.0 g/100 g of fresh carrot pulp significantly influenced the b* coordinate value of pellets and snacks, respectively. Differences in color coordinates showed the unambiguous effects of screw speeds applied during the processing of samples, with the same amount of vegetable pulp added.

### 3.3. Pasting Characteristics

**Figure 4:**
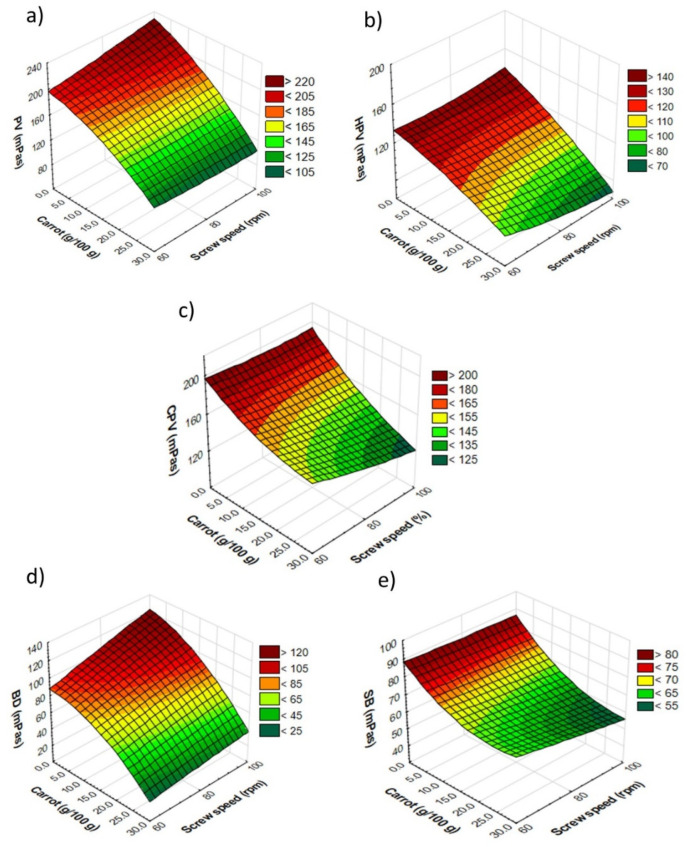
Results of pasting properties of extruded pellets enriched with fresh carrot pulp: (**a**) PV—peak viscosity, (**b**) HPV—hot paste viscosity, (**c**) CPV—cold paste viscosity, (**d**) BD—breakdown, (**e**) SB—setback.

Peak viscosity (PV) is closely related to the degree of starch granule damage, with severe damage directly leading to a higher peak viscosity [33]. The PV for pellets ranged from 110 mPas (maximum additive content, extruded at 80 rpm) to 223.5 mPas (2.5 g/100 g additive, processed at maximum speed). In general, along with the increase in the fresh carrot pulp addition in the pellets, there was a decrease in the PV values compared to the control sample (processed at 60 rpm) at the level of 20.0 and 30.0 g/100 g of this additive for all levels of additives at 80 rpm and above 2.5 g/100 g, when the maximum rpm was applied during processing. An increase in pellet PV was observed from 0 to 5.0 g/100 g of additive with increasing screw speed (Figure 4a).

**Figure 5:**
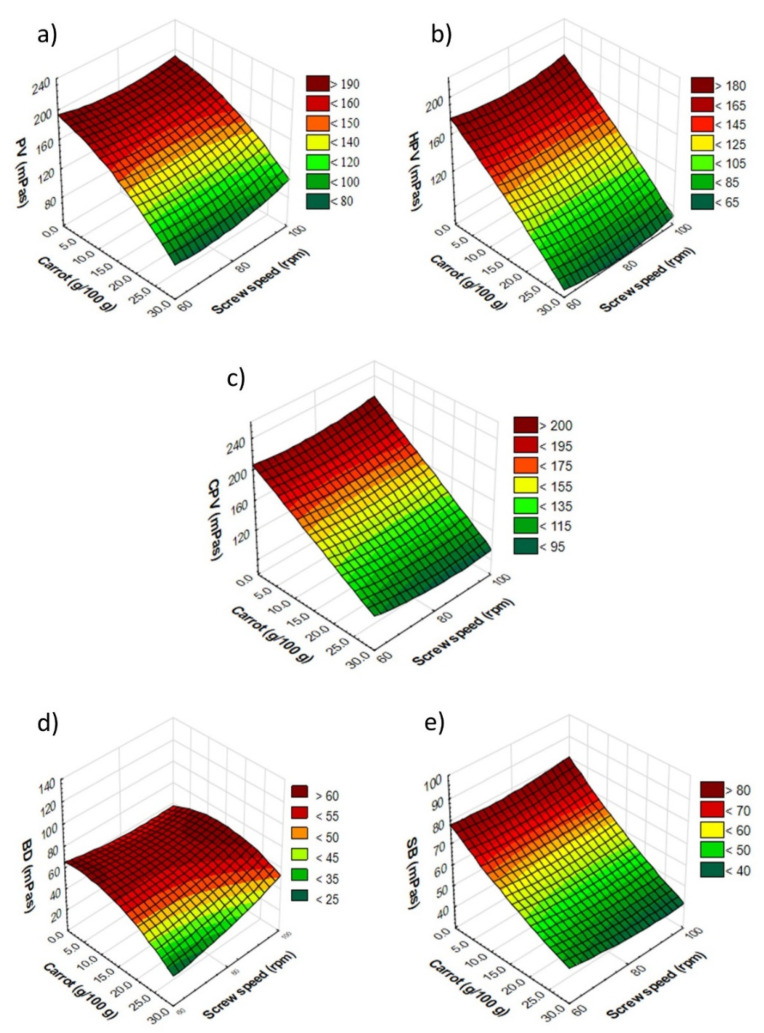
Results of pasting properties of expanded snacks enriched with fresh carrot pulp: (**a**) PV—peak viscosity, (**b**) HPV—hot paste viscosity, (**c**) CPV—cold paste viscosity, (**d**) BD—breakdown, (**e**) SB—setback.

The pellet PV was strongly negatively correlated with the amount of additive (r = −0.84), the AA and the a* color coordinate of pellets (r = −0.82 and −0.83), and strongly positively correlated with the VEI (r = 0.83), HPV (r = 0.94), CPV (r = 0.81) and BD (r = 0.97) of pellets, and PV (r = 0.84), HPV (r = 0.91), CPV (r = 0.90) and SB (r = 0.83) of the expanded snacks. The lowest PV value was 85 mPas, and was observed for snacks made from pellets with the lowest PV. The highest PV value (201 mPas) was recorded for the snack with 5.0 g/100 g pulp content processed at the lowest screw speed. For RTE snacks, the lowest PV values were recorded for samples produced at 80 rpm, with a fresh carrot level of 5.0 g/100 g and above (Figure 5a). The PV of snacks was strongly negatively correlated with the amount of additive (r = −0.94), the TPC of pellets (r = −0.86) and snacks (r = −0.85), the AA of pellets (r = −0.88) and snacks (r = −0.86), the a* coordinate of pellets (r = −0.91) and a* and b* of expanded snacks (r = −0.84 and −0.85, respectively). Negative correlation was noted between the PV of snacks and the VEI (r = 0.82), pellet lightness (r = 0.81), HPV (r = 0.91), CPV (r = 0.87), and snacks SB (r = 0.85).

Hot paste viscosity (HPV) indicates the sensitivity of the swollen starch granules to the shearing forces. The HPV values were within the range of from 77 mPas (pellets with the maximum additive content, processed at the maximum rpm) to 163 mPas (pellets with 10.0 g/100 g addition, extruded at 60 rpm) for carrot-supplemented pellets (Figure 4b). HPV was also strongly positively correlated with pellet CPV (r = 0.89), BD (r = 0.86) and SB (r = 0.81), and with supplemented snack HPV (r = 0.86) and CPV (r = 0.86). The HPV of microwave-expanded snacks was similar, and ranged from 65 mPas (maximum additive content, extruded at 80 rpm) to 172 mPas (control sample, made at 60 rpm) (Figure 5b).

In RTE directly expanded crisps, the HPV values of 18 to 38 mPas were observed for white bean extrudates, and 27 to 59 mPas for red bean extrudates [21]. The HPV of snacks was strongly negatively correlated with the amount of fresh carrot amount (r = −0.95), the TPC of pellets (r = −0.89) and snacks (r = −0.81), the AA of pellets (r = −0.92) and snacks (r = −0.85), the a* coordinate of pellets (r = −0.92), and a* (r = −0.87) and b* (r = −0.85) of the expanded snacks. A positive correlation was found between the HPV of snacks and CPV (r = 0.87), BD (r = 0.83) and SB (r = 0.82) of pellets, and between the HPV of snacks and CPV (r = 0.98) and SB (r = 0.94) of RTE snacks.

CPV denotes the final viscosity, indicating a tendency to the retrogradation of soluble amylose after cooling [34]. The CPV for pellets ranged from 122 mPas (pellets with the lowest HPV) to 200 mPas (pellets with a 2.5 g/100 g addition to the recipe, extruded at 60 rpm). In the case of the CPV measured for pellets, a decrease was noticed with the increasing screw speed applied, except for the control sample and the sample with 20.0 g/100 g of fresh carrot pulp in its composition (Figure 4c). The pellet CPV was strongly negatively correlated with the amount of additive (r = −0.84) and pellet and snack TPC (r = −0.86 and −0.85, respectively), and the a* coordinate of snacks (r = −0.83). A positive correlation was observed between the CPV of pellets and their SB parameters (r = 0.97). The CPV for snacks ranged from 99.5 mPas (snacks with the lowest PV and HPV) to 210 mPas (control snacks, at 100 rpm). In the case of microwave-expanded snacks, the value of this parameter decreased above 40% for the samples with 20 g/100 g of the vegetable component at all the rpms used, as compared to the control sample (Figure 5c). The snack CVP was strongly negatively correlated with the amount of additive (r = −0.93), pellet TPC (r = −0.89), pellet and snack AA (r = −0.91 and −0.84, respectively), the a* coordinate of pellets (r = −0.91), and a* (r = −0.84) and b* of snacks (r = −0.80). A strong positive correlation was found between the CPV of snacks and the VEI (r = 0.80), BD (r = 0.83), and the SB of pellets (r = 0.82) and snacks (r = 0.97).

Breakdown (BD) is related to the resistance to heating and shearing stress. It is also an indicator of swollen starch granule disintegration [35]. The lowest BD value was recorded for pellets (18.5 mPas) and snacks (19.5 mPas) with the maximum additive content, processed at the minimum rpm. In contrast, the highest value of BD was 132 mPas and 88.5 mPas, respectively, for pellets with 2.5 g/100 g of additive and snacks with a 20.0 g/100 g addition of carrot pulp, both processed at the highest rpm. Microwave expansion decreased the BD value of carrot-supplemented snacks to a level of 10 g/100 g at all rpms used, compared to the pellets (Figure 4d and Figure 5d). Moreover, pellet BD was strongly correlated with pellet bulk density (r = 0.82) and with pellet VEI (r = 0.84).

Setback (SB) shows the retrogradation behavior of starch gels. A high value of SB indicates a greater tendency to retrogradation, and thus translates into a greater probability of syneresis in the product [21]. The highest SB value was 90 mPas (pellets with 2.5 g/100 g additive content, extruded at 60 rpm) and 83 mPas (control snack made at 100 rpm). While the lowest SB value was recorded for pellets and snacks with the maximum fresh carrot pulp content when processed at maximum screw speed (57 mPas and 42.5 mPas, respectively). Generally, microwave expansion resulted in lower SB values of the expanded snacks in relation to pellets (Figure 4e and Figure 5e). In our study, we noticed that the pellet SB was strongly negatively correlated with the amount of additive (r = −0.84), the pellet and snack TPC (r = −0.87 and −0.81, respectively), the AA of snacks (r = −0.82), and the a* coordinate of the expanded snacks (r = −0.85). The SB of pellets also showed a strong positive correlation with the lightness of snacks (r = 0.83) and snack SB (r = 0.84). Similarly, the SB of carrot-supplemented snacks showed a strong negative correlation with the amount of additive (r = −0.93), the pellet and snack TPC (r = −0.92 and −0.83, respectively), pellet and snack AA (r = −0.89 and −0.88, respectively), a* coordinate of pellets (r = −0.90), and a* (r = −0.84) and b* (r = −0.81) of microwaved snacks.

### 3.4. Texture of Extrudates and Final RTE Products

Textural features play a key role in assessing snack quality and are also related to consumer acceptability [36]. Table 4 shows the values of the pellet cutting force and the results of texture tests for microwave-expanded snacks supplemented with fresh carrot pulp. Pellets with 2.5 g/100 g of carrots in the composition produced at 80 rpm showed the highest CF (88.92 N), while the lowest value of the tested parameter was characteristic for pellets (15.26 N) containing 30.0 g/100 g of the additive and produced at 60 rpm. This was due to the disruption of the internal structure by the vegetable component. It was noticed that the extreme tests of CF of the pellets differed significantly between each type and the control pellets obtained at the same speed.

Snacks expanded from these pellets also showed the lowest hardness (99.92 N) via the Kramer cell test. In addition, we saw that there was a positive correlation between the CF and the L* (r = 0.63) color coordinate. The pellets made at 100 rpm from the control recipe, and the samples with 2.5 g/100 g of the additive extruded at 60 rpm (195.40 N and 197.00 N, respectively) were the hardest. In a study of snacks with the addition of apple pomace above 28%, a decrease in the diameter of the air cells and an increase in the hardness of the formed snack were noted [37]. In our case, decreasing pellet hardness was the result of the decreasing amount of starch undergoing gelatinization, in favor of fresh carrot pulp. The extreme values of crispness were observed during the testing of the control sample. Here, the minimum value was observed at the lowest rpm (10.47 N)—indicating a good crispness value in the expanded snacks. Snacks made from the control blend were characterized by the highest crispness, as well as the fracturability, when extruded at 80 rpm (34.97 N and 37.58 N) and 100 rpm (29.52 N and 36.30 N), respectively. After microwave expansion, snacks supplemented with 7.5 g/100 g of fresh carrot pulp in the composition, processed at 100 rpm, showed the minimum fracture force value. Nevertheless, all snacks expanded by microwaves showed a narrow range of texture profile result variability, especially CR and FR, with insignificant effects of both the carrot pulp amount and the screw speed applied. Additionally, there was a positive correlation between the crispness and fracturability of snacks (r = 0.77). The higher amount of carrot in the recipe had an unambiguous effect on the crispness of RTE snacks.

### 3.5. Principal Component Analysis (PCA)

**Figure 6:**
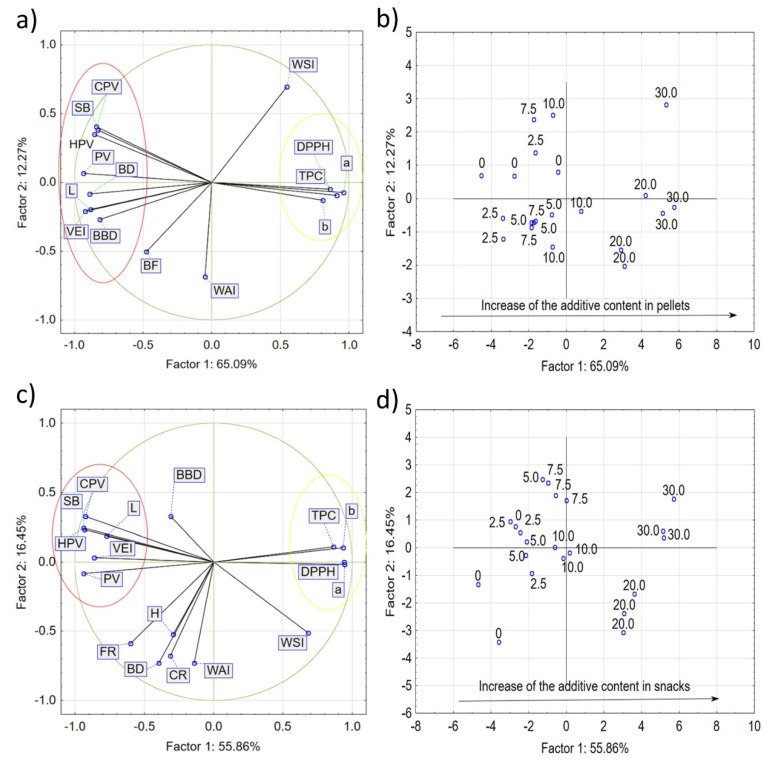
Analysis of principal components (PCA) of pellet (**a**) and snack (**c**) features and objects, as well as pellets (**b**) and snacks (**d**) in the space of the first two major components: TPC—total phenolic content; DPPH—antioxidant activity; BBD—bulk density; EI—volumetric expansion index; WAI—water absorption index; WSI—water solubility index; L*- lightness; a*—balance between redness (+) and greenness (-); b*—balance between blueness (-) and yellowness (+); PV—peak viscosity; HPV—hot paste viscosity; CPV—cold paste viscosity; BD—breakdown; SB—setback; CF—cutting force; H—hardness; CR—crispness; FR—fracturability; Factor 1—first component; Factor 2—second component; 0–30—amount of fresh carrot pulp (g/100 g).

Principal component analysis (PCA) is one of the most common statistical data reduction methods. Principal component analysis (Figure 6) was carried out in order to identify the differences in pellets and microwave-expanded snacks supplemented with fresh carrot pulp, processed at various rpm. PCA made it possible to select the discriminants that had the greatest impact on the differences between samples. The analysis itself consists of determining the component variables that are a linear combination of many examined features. The evaluation of the PCA graph (Figure 6a) shows that there is a strong but negative correlation between the parameters from the yellow ellipse and the parameters from the red ellipse. These parameters have the greatest influence on the system variability and describe the PC1 parameter. In the case of pellets, the first component of PC1 explained 65.09% of the variability of the results, and is positively related to TPC, AA, and the pellet a* and b* color coordinates. In contrast, it was negatively related to the BBD, the VEI, the lightness and the parameters describing pasting properties. In turn, the WAI and WSI are negatively correlated to each other. They also have a significant impact on the system variability and describe the second main component of PC2 in 12.27% of the results (highly negatively related to the WAI, and positively related to pellet WSI). There is no correlation between WAI and the parameters contained in the yellow and red ellipses. There is also a weak positive correlation between the WSI and the parameters in the yellow ellipse, and a weak negative correlation between the WSI and the parameters in the red ellipse. Additionally, there is a negative correlation between the WSI and CF. The location of the samples next to each other on the chart (Figure 6b,d) shows that the features are similar, and the distance between them indicates the nature of the differences. After placing the samples in the vicinity of the first two components (Figure 6b), it is evident that the samples differ from each other in terms of the carrot addition level. Moreover, significant differences can be seen at the supplementation levels of 20.0 and 30.0 g/100 g in relation to the lower levels of fortification. From this graph, it can be concluded that the first major component describes the system variability and determines the content of the additive to 65.09%. In the case of microwave-expanded snacks, the first component (Figure 6c) explains 55.86% of the variability of the results, and is positively related to TPC, AA, color coordinates a* and b* values, and negatively related to the snack VEI, L* and PV, HPV, CPV and SB. We can also conclude that there is a strong but negative correlation between the parameters from the yellow ellipse and the parameters from the red ellipse. The parameters contained in these ellipses have the greatest influence on the system variability when assessing the expanded snacks.

The second component accounts for 16.45% of the variability of the results and is highly negatively correlated with the expanded snack WAI and BD. Placing snacks results in the vicinity of the examined components (Figure 6d) showed generally similar relationships, as in the case of pellets—that snacks differ from each other with the increase in vegetable addition. However, greater differences were noticed between snacks containing 20.0 and 30.0 g/100 g of additive than between the corresponding pellets. From this graph, it can be concluded that the first major component describes the system variability and determines the effect of the vegetable additive to 55.86%. Here, PC1 takes positive values when the amount of fresh carrot pulp increases in the recipe.

## 4. Conclusions

The obtained results show that it is possible to acceptably enrich extruded snack pellets as half products with fresh carrot pulp, to the amount of up to 30.0 g/100 g. The RTE snacks obtained by microwave expansion of these pellets were characterized by a significant increase in the total content of polyphenols and by higher antioxidant activity, lower bulk density, higher WSI value, increased lightness, and decreased setback as compared to the pellets. The main impact on the differences between pellets and expanded snacks is shown by the measurements of the total polyphenol content, antioxidant activity, volumetric expansion index, color determinants and pasting properties (for snacks, except for breakdown). To sum up, a new type of microwave-expanded snack supplemented with the addition of fresh carrot pulp can be a high-quality, nutritionally valuable and fat-free alternative to the typically deep-fried crisps. Moreover, the knowledge from PCA analysis about the mutual influence of supplementation and screw speed during processing can be helpful in the design of new products processed by extrusion-cooking. The application of fresh vegetable material could not only be a valuable way to save water used in production and the energy needed for the drying process, but also have a positive effect on the material nutritional value, as compared to dried plant matter.

## Figures and Tables

**Table 1:** Total phenolic content and antioxidant activity of pellets and microwave-expanded snacks supplemented with fresh carrot pulp.

Amount of Carrot (g/100 g)	Screw Speed (rpm)	TPC ^1^ (mg GAE/100 g)		DPPH ^2^ (%)	
		P	S	P	S
0	60	58.88 ± 1.07 ^a^	127.52 ± 3.62 ^ab^	6.89 ± 0.56 ^a^	19.78 ± 0.34 ^a^
	80	61.65 ± 1.71 ^abc^	127.09 ± 3.20 ^ab^	6.61 ± 0.70 ^a^	19.74 ± 1.39 ^a^
	100	59.73 ± 0.64 ^ab^	124.54 ± 0.64 ^a^	6.65 ± 0.74 ^a^	19.50 ± 0.26 ^a^
2.5	60	61.86 ± 1.49 ^abcd^	128.37 ± 2.77 ^ab^	8.79 ± 0.91 ^ab^	23.31 ± 0.20 ^ab^
	80	62.72 ± 0.64 ^abcd^	129.23 ± 2.77 ^ab^	9.70 ± 0.70 ^ab^	25.29 ± 1.65 ^ab^
	100	64.21 ± 2.13 ^abcd^	130.29 ± 2.56 ^ab^	8.02 ± 0.28 ^ab^	24.88 ± 1.09 ^ab^
5.0	60	66.55 ± 0.21 ^abcdef^	130.72 ± 2.13 ^abc^	8.42 ± 0.72 ^ab^	23.72 ± 1.16 ^ab^
	80	66.34 ± 2.13 ^abcde^	133.28 ± 2.55 ^abc^	6.82 ± 0.35 ^a^	24.30 ± 0.58 ^ab^
	100	67.19 ± 1.28 ^bcdef^	134.56 ± 2.13 ^abc^	7.14 ± 0.32 ^a^	24.01 ± 0.29 ^ab^
7.5	60	69.54 ± 0.64 ^def^	136.05 ± 4.05 ^abc^	6.75 ± 0.56 ^a^	29.73 ± 0.89 ^bc^
	80	68.05 ± 0.85 ^cdef^	134.13 ± 2.56 ^abc^	6.47 ± 0.98 ^a^	30.17 ± 0.44 ^bcd^
	100	68.68 ± 0.21 ^cdef^	134.98 ± 2.13 ^abc^	7.63 ± 0.25 ^a^	30.06 ± 0.11 ^bcd^
10.0	60	74.23 ± 1.49 ^fgh^	133.92 ± 3.62 ^abc^	7.17 ± 0.56 ^a^	37.44 ± 0.82 ^ef^
	80	74.01 ± 0.85 ^efgh^	134.56 ± 2.12 ^abc^	7.67 ± 0.07 ^a^	36.83 ± 0.48 ^cde^
	100	72.95 ± 0.64 ^efg^	141.38 ± 1.71 ^abc^	7.07 ± 0.60 ^a^	36.95 ± 0.12 ^de^
20.0	60	78.92 ± 1.92 ^gh^	141.80 ± 3.84 ^abc^	11.32 ± 0.77 ^b^	43.52 ± 2.12 ^efg^
	80	79.34 ± 1.49 ^gh^	142.23 ± 2.98 ^bc^	19.67 ± 0.97 ^cd^	37.07 ± 0.24 ^de^
	100	80.41 ± 1.71 ^ghi^	140.31 ± 2.77 ^abc^	17.18 ± 0.67 ^c^	39.15 ± 2.20 ^ef^
30.0	60	81.48 ± 2.34 ^hij^	147.56 ± 1.92 ^cd^	22.90 ± 0.53 ^d^	49.48 ± 2.71 ^g^
	80	88.08 ± 0.43 ^ij^	160.56 ± 3.84 ^de^	20.17 ± 0.73 ^cd^	44.34 ± 1.02 ^fg^
	100	88.72 ± 1.07 ^j^	171.01 ± 4.05 ^e^	20.54 ± 0.37 ^dc^	47.27 ± 2.20 ^g^

TPC—total phenolic content; DPPH—antioxidant activity against DPPH radical; P—pellet; S—snack; ^1^—means of 3 replications, ^2^—means of 2 replications; ^a–j^—means indicated with similar letters in columns do not differ significantly at α = 0.05.

**Table 2:** Physical properties of pellets and microwave-expanded snacks with the addition of fresh carrot pulp.

Amount of Carrot (g/100 g)	Screw Speed (rpm)	WAI ^1^ (g/g)		WSI ^1^ (%)	
		P	S	P	S
0	60	4.53 ± 0.05 ^cdefgh^	4.82 ± 0.26 ^abc^	3.56 ± 0.01 ^abcdef^	4.32 ± 0.69 ^abcd^
	80	4.37 ± 0.06 ^cdef^	5.49 ± 0.14 ^cd^	2.76 ± 0.28 ^abcdef^	6.73 ± 0.35 ^defg^
	100	3.85 ± 0.04 ^ab^	5.00 ± 0.28 ^abcd^	2.78 ± 0.18 ^abcdef^	3.94 ± 0.32 ^abcd^
2.5	60	4.51 ± 0.10 ^cdefgh^	4.85 ± 0.29 ^abcd^	4.56 ± 0.24 ^fg^	5.54 ± 1.31 ^bcde^
	80	4.65 ± 0.02 ^defgh^	4.54 ± 0.16 ^a^	1.77 ± 0.34 ^ab^	3.27 ± 0.50 ^ab^
	100	4.29 ± 0.01 ^cde^	4.69 ± 0.23 ^ab^	1.99 ± 0.69 ^abc^	3.71 ± 0.23 ^abc^
5.0	60	4.76 ± 0.03 ^fgh^	4.72 ± 0.09 ^ab^	1.42 ± 0.35 ^a^	4.79 ± 0.06 ^abcd^
	80	4.90 ± 0.03 ^h^	4.53 ± 0.02 ^a^	1.91 ± 0.40 ^abc^	2.34 ± 0.05 ^a^
	100	4.67 ± 0.03 ^efgh^	5.43 ± 0.37 ^bcd^	1.85 ± 0.25 ^abc^	4.11 ± 0.10 ^abcd^
7.5	60	3.71 ± 0.10 ^a^	4.49 ± 0.02 ^a^	3.76 ± 0.52 ^cdefg^	3.92 ± 1.23 ^abcd^
	80	4.76 ± 0.33 ^fgh^	4.78 ± 0.21 ^abc^	2.83 ± 0.22 ^abcdef^	5.15 ± 0.90 ^abcde^
	100	4.66 ± 0.03 ^efgh^	4.98 ± 0.11 ^abcd^	2.55 ± 0.24 ^abcdef^	4.99 ± 0.20 ^abcd^
10.0	60	3.87 ± 0.06 ^ab^	4.70 ± 0.30 ^ab^	3.92 ± 1.10 ^cdefg^	5.97 ± 0.10 ^bcde^
	80	4.81 ± 0.01 ^gh^	4.79 ± 0.17 ^abc^	2.21 ± 0.06 ^abcd^	4.00 ± 0.82 ^abcd^
	100	4.85 ± 0.10 ^gh^	4.60 ± 0.04 ^a^	2.68 ± 0.34 ^abcdef^	4.10 ± 0.70 ^abcd^
20.0	60	4.25 ± 0.08 ^bcd^	5.05 ± 0.04 ^abcd^	4.10 ± 0.46 ^defg^	6.19 ± 0.19 ^cdef^
	80	4.34 ± 0.24 ^cde^	5.05 ± 0.14 ^abcd^	2.41 ± 0.69 ^abcde^	8.83 ± 0.24 ^fgh^
	100	4.55 ± 0.12 ^cdefgh^	5.61 ± 0.29 ^d^	2.70 ± 0.58 ^abcdef^	10.48 ± 1.57 ^h^
30.0	60	4.15 ± 0.05 ^bc^	4.45 ± 0.27 ^a^	5.69 ± 0.91 ^g^	7.85 ± 1.29 ^efgh^
	80	4.44 ± 0.14 ^cdefg^	4.70 ± 0.05 ^ab^	3.46 ± 1.27 ^abcdef^	9.28 ± 0.10 ^gf^
	100	4.32 ± 0.09 ^cde^	4.53 ± 0.15 ^a^	4.48 ± 0.27 ^efg^	5.34 ± 0.86 ^bcde^

WAI—water absorption index; WSI—water solubility index; P—pellet; S—snack; ^1^—means of 3 replications; ^a–h^—means indicated with similar letters in columns do not differ significantly at α = 0.05.

**Table 3:** The color of pellets and microwave-expanded snacks with the addition of fresh carrot pulp.

Amount of Carrot (g/100 g)	Screw Speed (rpm)	L* ^1^		a* ^1^		b* ^1^		*ΔE*	
		P	S	P	S	P	S	P	S
0	60	78.21 ± 1.09 ^defg^	84.03 ± 0.63 ^cdefg^	5.74 ± 0.29 ^abc^	8.43 ± 0.25 ^a^	26.51 ± 0.58 ^cd^	22.30 ± 0.41 ^ab^	ref	ref
	80	81.98 ± 1.78 ^ghijk^	85.50 ± 1.95 ^fg^	5.49 ± 0.25 ^abc^	8.17 ± 0.20 ^a^	24.40 ± 0.52 ^abc^	21.52 ± 0.50 ^a^	ref	ref
	100	85.06 ± 2.52 ^k^	86.03 ± 1.00 ^fg^	5.07 ± 0.38 ^ab^	8.36 ± 0.10 ^a^	22.22 ± 0.76 ^a^	21.95 ± 0.19 ^ab^	ref	ref
2.5	60	81.67 ± 0.75 ^ghijk^	85.93 ± 1.45 ^fg^	5.64 ± 0.18 ^abc^	8.44 ± 0.11 ^a^	25.79 ± 0.60 ^bcd^	22.93 ± 0.35 ^bc^	0.36	2.01
	80	84.33 ± 1.25 ^jk^	85.13 ± 1.16 ^efg^	4.81 ± 0.18 ^a^	8.62 ± 0.19 ^ab^	23.61 ± 0.41 ^ab^	22.90 ± 0.31 ^bc^	0.54	1.50
	100	80.81 ± 1.37 ^fghij^	86.84 ± 1.34 ^g^	6.20 ± 0.43 ^bcd^	8.72 ± 0.25 ^ab^	26.27 ± 0.93 ^bcd^	23.12 ± 0.68 ^bcd^	1.17	1.46
5.0	60	80.31 ± 0.53 ^efghi^	84.81 ± 0.89 ^defg^	6.54 ± 0.17 ^cd^	9.10 ± 0.21 ^bc^	27.28 ± 1.02 ^d^	23.79 ± 0.42 ^cde^	0.72	1.81
	80	77.27 ± 0.54 ^cdef^	84.44 ± 1.71 ^defg^	8.47 ± 0.18 ^ef^	9.51 ± 0.25 ^cde^	33.04 ± 1.38 ^fgh^	24.45 ± 0.39 ^ef^	1.52	3.39
	100	78.80 ± 0.51 ^defg^	85.67 ± 0.73 ^fg^	8.11 ± 0.25 ^ef^	9.37 ± 0.21 ^cd^	30.64 ± 0.29 ^ef^	24.24 ± 0.34 ^de^	2.06	2.52
7.5	60	80.23 ± 1.36 ^efghi^	85.09 ± 0.32 ^efg^	7.42 ± 0.32 ^de^	9.16 ± 0.09 ^bc^	26.28 ± 0.80 ^bcd^	24.20 ± 0.16 ^de^	0.35	2.30
	80	83.06 ± 0.75 ^ijk^	83.98 ± 1.38 ^bcdef^	7.29 ± 0.35 ^de^	10.53 ± 0.19 ^fg^	27.90 ± 1.00 ^de^	26.11 ± 0.49 ^gh^	1.14	5.75
	100	82.74 ± 2.77 ^hijk^	82.97 ± 0.84 ^bcdef^	7.43 ± 0.55 ^de^	9.42 ± 0.19 ^cd^	27.48 ± 0.80 ^d^	23.82 ± 0.51 ^cde^	0.30	3.74
10.0	60	77.57 ± 0.36 ^cdef^	82.16 ± 0.76 ^abcde^	8.88 ± 0.16 ^fg^	9.84 ± 0.07 ^de^	31.35 ± 0.84 ^fg^	24.09 ± 0.10 ^cde^	0.78	2.94
	80	76.13 ± 0.97 ^cd^	83.64 ± 1.11 ^cdef^	10.04 ± 0.62 ^g^	10.02 ± 0.19 ^ef^	31.27 ± 0.74 ^fg^	24.88 ± 0.43 ^efg^	0.92	4.26
	100	81.00 ± 1.40 ^fghij^	81.72 ± 0.96 ^abcd^	8.21 ± 0.70 ^ef^	9.93 ± 0.29 ^de^	25.79 ± 2.57 ^bcd^	23.88 ± 0.58 ^cde^	2.16	4.97
20.0	60	76.59 ± 1.96 ^cde^	79.41 ± 0.52 ^a^	12.43 ± 1.30 ^hi^	10.99 ± 0.26 ^gh^	33.43 ± 1.77 ^fg^	25.64 ± 0.67 ^fg^	1.79	6.25
	80	78.89 ± 0.56 ^defgh^	79.98 ± 1.37 ^ab^	11.74 ± 0.53 ^h^	11.05 ± 0.14 ^ghi^	31.97 ± 0.70 ^fgh^	26.14 ± 0.39 ^gh^	1.27	7.76
	100	76.85 ± 1.64 ^cde^	82.08 ± 0.68 ^abcde^	12.58 ± 0.46 ^hi^	11.45 ± 0.13 ^hij^	32.93 ± 0.74 ^fgh^	27.18 ± 0.42 ^hi^	0.88	7.24
30.0	60	70.46 ± 2.25 ^ab^	82.08 ± 1.09 ^abcde^	13.39 ± 0.76 ^ij^	11.75 ± 0.43 ^j^	30.75 ± 1.20 ^fg^	27.48 ± 1.15 ^i^	1.40	6.45
	80	69.23 ± 1.29 ^a^	83.45 ± 1.82 ^cdef^	16.09 ± 0.39 ^g^	10.88 ± 0.04 ^gh^	34.26 ± 0.71 ^h^	26.93 ± 0.22 ^hi^	0.55	6.39
	100	73.95 ± 2.33 ^bc^	81.06 ± 1.97 ^abc^	14.62 ± 0.69 ^j^	11.59 ± 0.45 ^ij^	32.42 ± 1.52 ^fgh^	27.29 ± 0.52 ^hi^	0.85	7.97

L*—lightness from 0 to 100; a*—balance between redness (+) and greenness (-); b*—balance between blueness (-) and yellowness (+); *ΔE*—total color change index; P—pellet; S—snack; ^1^—means of 10 replications; ^a–k^—means indicated with similar letters in columns do not differ significantly at α = 0.05.

**Table 4:** Cutting force of pellets and the texture profile of microwave-expanded snacks with the addition of fresh carrot pulp.

Amount of Carrot (g/100 g)	Screw Speed (rpm)	CF of Pellets ^1^ (N)	Texture of Expanded Snacks		
			H ^2^ (N)	CR ^2^ (N)	FR ^2^ (N)
0	60	43.70 ± 7.57 ^bcd^	170.40 ± 15.96 ^abc^	10.47 ± 1.50 ^a^	26.07 ± 4.96 ^ab^
	80	55.22 ± 7.72 ^de^	158.00 ± 14.06 ^abc^	34.97 ± 5.62 ^b^	37.58 ± 6.76 ^b^
	100	66.38 ± 7.96 ^efg^	195.40 ± 19.17 ^c^	29.52 ± 5.45 ^ab^	36.30 ± 6.12 ^b^
2.5	60	75.64 ± 5.95 ^gh^	197.00 ± 20.97 ^bc^	20.88 ± 3.69 ^ab^	23.88 ± 5.66 ^ab^
	80	88.92 ± 6.50 ^h^	149.60 ± 14.84 ^abc^	18.34 ± 3.81 ^ab^	25.76 ± 5.97 ^ab^
	100	83.28 ± 8.46 ^gh^	162.20 ± 18.41 ^abc^	16.13 ± 3.29 ^ab^	26.34 ± 3.90 ^ab^
5.0	60	56.06 ± 6.40 ^def^	150.40 ± 14.39 ^abc^	18.51 ± 4.93 ^ab^	26.52 ± 4.81 ^ab^
	80	43.64 ± 6.61 ^bcd^	136.16 ± 17.41 ^ab^	13.81 ± 2.66 ^ab^	19.61 ± 2.37 ^ab^
	100	45.56 ± 3.88 ^bcd^	109.40 ± 4.45 ^a^	21.38 ± 5.24 ^ab^	25.56 ± 4.94 ^ab^
7.5	60	35.00 ± 5.18 ^bc^	109.98 ± 16.38 ^a^	16.26 ± 2.02 ^ab^	19.00 ± 3.46 ^ab^
	80	53.18 ± 5.69 ^de^	107.38 ± 15.85 ^a^	18.82 ± 2.47 ^ab^	23.62 ± 4.45 ^ab^
	100	53.64 ± 7.16 ^de^	126.82 ± 12.90 ^ab^	13.57 ± 2.75 ^ab^	14.67 ± 2.95 ^a^
10.0	60	45.74 ± 5.57 ^bcd^	192.00 ± 28.01 ^abc^	17.55 ± 4.10 ^ab^	22.77 ± 2.26 ^ab^
	80	29.42 ± 4.22 ^ab^	164.60 ± 19.21 ^abc^	18.67 ± 4.59 ^ab^	25.43 ± 6.69 ^ab^
	100	55.48 ± 6.16 ^de^	157.58 ± 20.47 ^abc^	21.50 ± 5.48 ^ab^	28.72 ± 5.91 ^ab^
20.0	60	32.64 ± 5.82 ^bc^	168.20 ± 23.68 ^abc^	24.50 ± 3.5 ^ab^	25.48 ± 4.46 ^ab^
	80	73.12 ± 6.94 ^fgh^	151.20 ± 37.98 ^abc^	18.49 ± 2.45 ^ab^	18.94 ± 2.28 ^ab^
	100	81.24 ± 9.28 ^gh^	143.40 ± 32.32 ^abc^	17.17 ± 5.9 ^ab^	24.25 ± 4.23 ^ab^
30.0	60	15.26 ± 3.20 ^a^	99.92 ± 20.42 ^a^	15.88 ± 4.73 ^ab^	16.23 ± 4.48 ^ab^
	80	33.56 ± 6.33 ^bc^	161.40 ± 30.76 ^abc^	16.26 ± 4.9 ^ab^	18.45 ± 4.09 ^ab^
	100	47.16 ± 7.19 ^cd^	141.20 ± 32.93 ^abc^	16.64 ± 5.99 ^ab^	23.42 ± 4.14 ^ab^

CF—cutting force; H—hardness; CR—crispness; FR—fracturability; P—pellet; S—snack; ^1^—means of 10 replications; ^2^—means of 5 replications; ^a–h^—means indicated with similar letters in columns do not differ significantly at α = 0.05.

## Data Availability

The data presented in this study are available on request from the corresponding author.
